# The 2019 Universiti Teknologi MARA, Malaysia Staff Survey: Determining the Level and Predictors of Quality of Life

**DOI:** 10.3389/fpsyt.2021.705018

**Published:** 2021-08-09

**Authors:** Mohd Izwan Mat Nazali, Salmi Razali, Suthahar Ariaratnam, Yuhaniz Ahmad, Hapizah Nawawi

**Affiliations:** ^1^Department of Psychiatry, Faculty of Medicine, Selangor Campus, Universiti Teknologi MARA, Shah Alam, Malaysia; ^2^Institute for Pathology, Laboratory, and Forensic Medicine (I-PPerForM), Shah Alam, Malaysia; ^3^Department of Psychiatry, Faculty of Medicine, Universiti Teknologi MARA, Cawangan Selangor, Kampus Selayang, Shah Alam, Malaysia; ^4^School of Quantitative Sciences, Universiti Utara Malaysia, Changlun, Malaysia; ^5^I-PPerForM (Institute of Pathology, Forensic and Laboratory Medicine) and Faculty of Medicine, Universiti Teknologi MARA, Sungai Buloh, Malaysia

**Keywords:** quality of life, university staff, depression, job satisfaction, predictors

## Abstract

Experiencing good quality of life (QOL) among university staff is extremely crucial to ensuring academic excellence; however, there are limited data on factors that contribute to QOL among university staff. This study aims to determine the level and the predictors for good QOL among university staff. The consenting participants were selected using a stratified sampling method. Participants who had fulfilled the selection criteria were provided with socio-demographic, medical illness, job factor, and family background questionnaires. QOL and psychological well-being (depression, anxiety, and stress) were assessed using the World Health Organization Quality of Life brief version (WHOQOL-BREF) and Depression, Anxiety, and Stress Scale (DASS-21) questionnaires, respectively. A total of 278 staff (mean ± SD age: 38.84 ± 7.85 years, 44.2% males, 82.7% married) had participated in this study. This study found that participants had low QOL in the domains of physical health [P-QOL] (11.2%), psychological health [PSY-QOL] (9.7%), social relationships [SR-QOL] (19.1%), and environment [E-QOL] (14.4%). The predictors of P-QOL were depression, medical illness, and number of dependents, while those of PSY-QOL were work promotion, depression, medical illness, and number of dependents. Additionally, the predictors of SR-QOL were campus location, depression, and work promotion, while those of E-QOL were age, level of education, depression, work promotion, and medical illness. Depression significantly affected all domains of QOL. Younger participants without medical illness and those with tertiary level of education had increased odds of having good QOL. Participants having dependents without work promotion and employed in suburban areas had decreased odds of having good QOL. The relevant authority should be identified and then assist staff with difficulties to ensure the staff benefited from having a good QOL.

## Introduction

Quality of life (QOL) is a diverse, complex, and multidimensional concept that includes subjective assessment of both positive and negative aspects of one's life (1, 2). Given its complexity and lack of universal definition and measure, various researchers of different backgrounds have attempted to define as well as conceptualize QOL throughout the years (1, 3–5). The World Health Organization (WHO) has defined QOL as “an individual's perception of their position in life in the context of the culture and value systems in which they live and in relation to their goals, expectations, standards, and concerns” (1, 2).

QOL comprises numerous domains, such as physical, psychological, social, and environmental (1). It affects various aspects of one's life and contributes significantly to one's perception of well-being, health, happiness, and life satisfaction (2, 6). From a healthcare perspective, the increasing knowledge on QOL had been recognized as an essential tool in assessing health outcomes, thereby aiding crucial decisions in healthcare policy and preventive medicine (7, 8). On a larger scale, QOL among various countries' populations was positively associated with a country's human development index (HDI) (9). With the current knowledge of how QOL affects a person in general, it is vital to ensure good QOL among university staff too. Additionally, it helps to maintain motivation, life satisfaction, and job satisfaction and reduce stress as well as burnout among staff (10, 11).

Studies in the general population had shown that factors and predictors such as older age (12), depression (13), anxiety (14–17), stress (18), chronic medical illness (19–23), poor financial status (24–26), low level of education (27, 28), being single (29), family problem (30–32), and poor job satisfaction (33, 34) may negatively affect QOL (35, 36). However, there are limited data available pertaining to QOL among university staff.

Globally, pertaining to staff and workers, most studies have focused on assessing quality of work life (QWL) instead of QOL (37–39). Furthermore, QWL focused mainly on personal reactions toward the working environment rather than one's life as a whole (33, 40, 41). Another study found that burnout among faculty staff negatively impacted QOL, regardless of participants' field of knowledge (11). A survey that was conducted on 522 staff of Neyshabur healthcare centers revealed that chronic illness in healthcare workers was affecting QOL (42). Researchers have also explored QOL among university employees, but it was confined to academicians (41). In a study conducted locally on QOL among university staff, only association with physical activity was investigated, and regrettably no determining predictors were found (43).

Thus, the objective of the present study was to determine the level and predictors of QOL among academic and non-academic staff at the Universiti Teknologi MARA (UiTM), Malaysia. Specifically, the study investigated the association of QOL with socio-demographic factor, job factor, psychological well-being, physical condition, and family background. Accordingly, the policymakers, specifically university administrators, would benefit from the information gathered, which would aid them in future planning and resource allocation. Moreover, this would surely help in early detection, mental health promotion, and provision of assistance for those affected by low QOL. To the best of our knowledge, this is the first study investigating the link between QOL and various factors such as psychological well-being, job factor, and family background among both academic and non-academic university staff.

## Methods

This was a cross-sectional study conducted from January 2019 to December 2019, involving selected participants of UiTM aged between 18 and 70 years. UiTM is a public university based primarily in Shah Alam, Malaysia. It has since grown into the largest institution of higher education in Malaysia as measured by physical infrastructure, number of faculties, staff, and student enrolments. The university is composed of one main campus and 34 satellite campuses. It offers over 500 programs that range from undergraduate to postgraduate levels.

**Figure 1:**
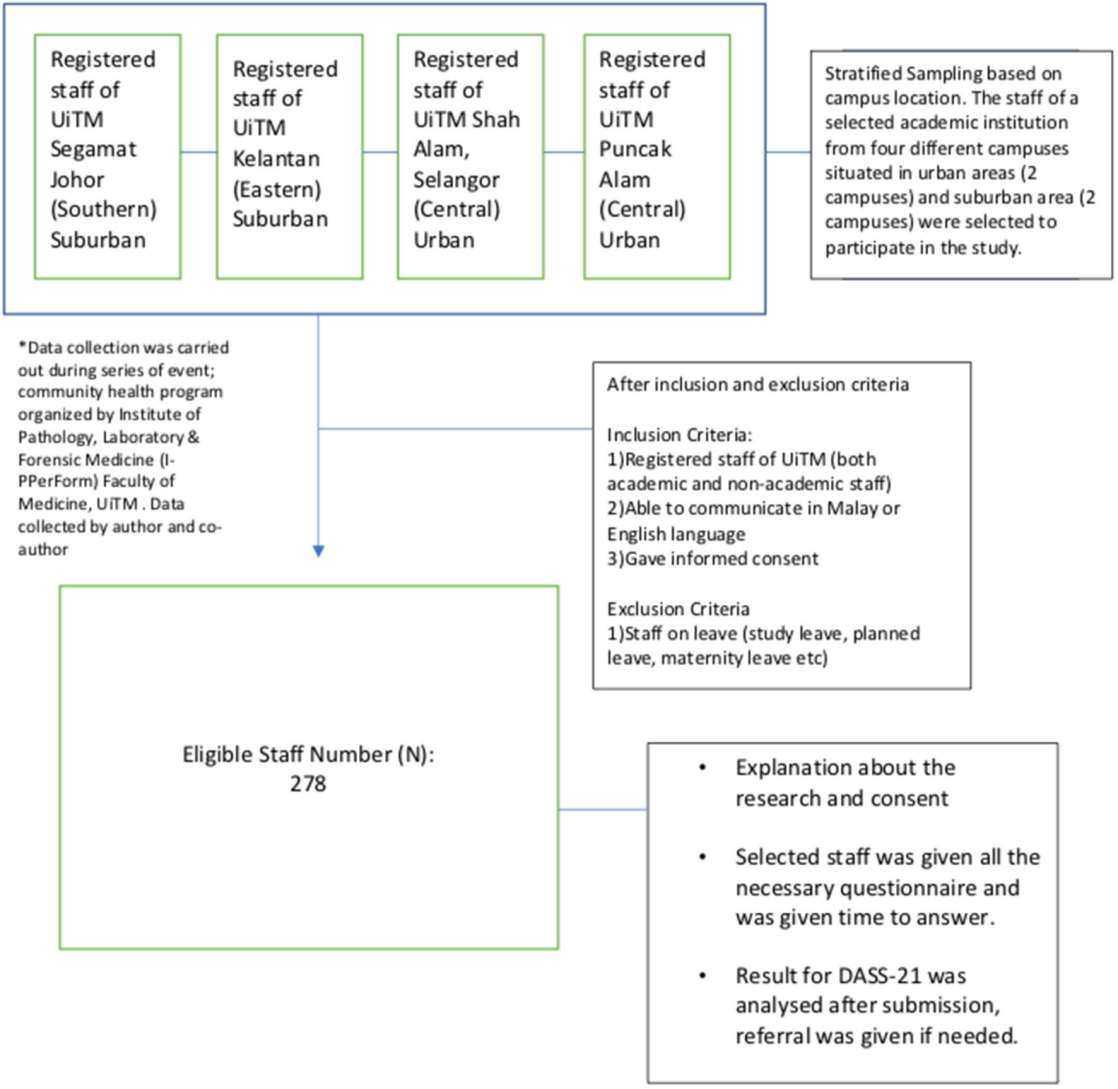
Flow chart of the technique used in selecting participants.

The inclusion criteria included registered staff of UiTM (both academic and non-academic), who were able to communicate in Malay or English languages and provided informed consent. The exclusion criteria included staff who were on leave. A stratified sampling method was used based on the locations of UiTM campuses, which were either urban or suburban. Staff of a selected academic institution, hailing from four different campuses that were situated in urban areas (comprising two campuses) and suburban areas (comprising two campuses), were selected to participate in the study (refer to Figure 1).

The sample size was calculated using the single proportion formula with 5% precision and 95% confidence interval (CI) from a total population of 17,700, resulting in 278 participants. Ethical approval was obtained from the Institutional Ethics Committee (600-IRMI-5/1/6-REC/398/18).

Participants were required to complete the socio-demographic data (consisting of gender, age, ethnicity, marital status, and educational level), job factor particulars (comprising household income in a month, campus location, current years of service duration, work promotion, and job satisfaction), details of physical condition (consisting of preexisting medical illness such as diabetes mellitus, hypertension, hyperlipidemia, or other non-communicable diseases), and family background characteristics (such as number of dependents, family problem, and workplace location). The definition of an urban campus was provided by the Department of Statistic, Malaysia. Based on the socioeconomic status in Malaysia, three different income groups have emerged, namely, top 20% (T20) [income ranged above Ringgit Malaysia (RM) 10,960], middle 40% (M40) [income ranged between RM 4850 and RM 10,959], and bottom 40% (B40) [income ranged below RM 4849] (44). Incidentally, the exchange rate is RM 4.15 to US$1.

The psychological well-being was measured using the Malay version of Depression, Anxiety, Stress Scale (BM-DASS-21) (45). BM-DASS-21 is a self-report questionnaire with good internal reliabilities with Cronbach's alpha of 0.79 for stress. It has been used in many studies involving academic staff in this country (46). Participants were asked to rate their experience on each symptom over the past week on a 4-point severity scale ranging from 0 (does not apply to me) to 3 (applies to me most of the time). Scores for each scale were later summed up and categorized as normal, mild, moderate, severe, and extremely severe. For depression, the total scoring was categorized as follows: normal (0–9), mild (10–13), moderate (14–20), severe (21–27), and extremely severe (more than 28). For anxiety, the total scoring was categorized as normal (0–7), mild (8–9), moderate (10–14), severe (15–19), and extremely severe (more than 20). For stress, the total scoring was categorized as normal (0–14), mild (15–18), moderate 19–25), severe (26–33), and extremely severe (34 and above). Participants who have normal score were considered normal while those who have mild, moderate, severe, and extremely severe varieties were deemed to have either depression, anxiety, or stress.

QOL was measured using the validated World Health Organization Quality of Life, brief version (WHOQOL-BREF). WHOQOL-BREF has good internal reliability with a Tucker–Lewis index (TLI) of 0.909, Cronbach's alpha exceeding 0.7, and intra-class correlation coefficient (ICC) exceeding 0.4 for the Malay version (47). It has been widely used in many studies across Malaysia (48–50). The domain score obtained was then transformed to a 0–100 scale (1), in which the result from the score was divided into low QOL and good QOL based on the calculation of each domain score. We defined one standard deviation (SD) score below the mean as the cutoff point for low QOL (51). The four domains of WHOQOL-BREF were physical [P-QOL], psychological health [PSY-QOL], social relationships [SR-QOL], and environment [E-QOL].

The data were analyzed using the Statistical Package for Social Sciences (SPSS) version 25.0 (IBM). Variables were described as mean ± SD for continuous data as well as number (*n*) and percentage (%) for dichotomous or nominal data. Factors associated with QOL were analyzed by simple logistic regression (SLogR) followed by multiple logistic regression (MLogR). The socio-demographic factors (gender, age, ethnicity, marital status, and educational level), psychological well-being (depression, anxiety, and stress), job factor particulars (household income, campus location, duration of service, work promotion, and job satisfaction), being the independent variables, were entered into the SLogR. Variables having a *p*-value of <0.05 from the SLogR were subsequently included in the MLogR analysis. Model fitness was checked using the Hosmer–Lemeshow goodness-of-fit test. Confounders were adjusted. Interactions, multicollinearity, and assumptions were also checked. A *p*-value of <0.05 with a CI of 95% was taken as statistically significant.

## Results

### Socio-Demographic and Other Characteristics of the Study Participants

A total of 278 participants were successfully recruited in this study. Table 1 shows the socio-demographic data, job factor particulars, psychological well-being, physical condition (i.e., medical illness), and family characteristics of the participants. More than half of the participants were female (*n* = 155; 55.8%) with a mean age of 38.91 ± 7.94 years. Majority of them were non-academician (*n* = 186; 66.9%), <45 years old (*n* = 185; 66.5.5%), Malays (*n* = 274; 98.6%), receiving tertiary education (*n* = 198; 71.2%), and married (*n* = 230; 82.7%).

**Table 1:** Sociodemographic, job factor, psychological well-being, physical condition and family characteristics of the participants.

**Variables**	**Frequency (*N*)**	**Percentage (%)**	***P*** **-Value**			
			**P-QOL**	**PSY-QOL**	**SR-QOL**	**E-QOL**
**Socio-demographic**						
Age range (years)			0.580	0.383	0.227	0.051
<45 years old	185	66.5				
>45 years old	93	33.5				
Gender			0.913	0.982	0.634	0.428
Male	123	44.2				
Female	155	55.8				
Ethnicity			0.475	0.298	0.328	0.409
Malay Non-Malay	274 4	98.6 1.5				
Level of education			0.195	0.429	0.206	0.188
Non-tertiary education	80	37.8				
Tertiary education: College or university	198	71.2				
Marital Status			0.406	0.004^*^	0.018^*^	0.162
Single	40	14.4				
Married	230	82.7				
Divorced	8	2.9				
**Psychological well-being**						
Depression			<0.001^*^	<0.001^*^	<0.001^*^	<0.001^*^
Normal	164	59				
Mild to extremely severe	114	41				
Anxiety			0.004^*^	0.005^*^	0.004^*^	0.005^*^
Normal	111	39.9				
Mild to extremely severe	167	60.1				
Stress			0.003^*^	<0.001^*^	0.109	0.014^*^
Normal	198	71.2				
Mild to extremely severe	80	28.8				
**Job factor**						
Job group			0.360	0.405	0.410	0.416
Academician	92	33.1				
Non-academician	186	66.9				
Household income in a month			0.636	0.879	0.156	0.275
< RM 10,960 (B40 and M40)	234	84.2				
≥ RM 10,960 (T20)	44	15.8				
Campus location			0.306	0.086	0.103	0.990
Urban	174	62.6				
Suburban	104	37.4				
Work promotion			0.166	0.003^*^	0.008^*^	<0.001^*^
Yes	175	62.9				
No	103	37.1				
Current years of service duration			0.947	0.215	0.264	0.772
<10 years	154	55.4				
≥10 years	124	44.6				
Job satisfaction			<0.001^*^	<0.001^*^	0.002^*^	0.001^*^
Satisfied	261	93.9				
Not satisfied	17	6.1				
**Physical condition**						
Having medical illness			0.002^*^	0.005^*^	0.310	0.467
Yes	64	23				
No	214	77				
**Family background**						
At least 1 dependent			0.80	<0.001^*^	0.082	0.257
Yes	214	77				
No	64	23				
Family Problem/Issue			0.965	0.939	0.668	0.871
Yes	225	80.9				
No	53	19.1				
Workplace far from family			0.711	0.286	0.134	0.694
Yes	180	64.7				
No	98	35.3				

* *Statistically significant at the 0.05 level*.

Psychological well-being profiling established that 114 (41%) had mild to extremely severe symptoms of depression, 80 (28.8%) had mild to extremely severe symptoms of stress, and 167 (60.1%) had mild to extremely severe symptom of anxiety.

For job factor domain, majority of the participants were from the low-income group (B40) and middle-income group (M40) (*n* = 234; 84.2%), worked in urban campuses (*n* = 174;62.6%), had been promoted (*n* = 175; 62.9%), had served <10 years in the current establishment (*n* = 154; 55.4%), and were satisfied with their current job (I = 261; 93.9%).

In terms of physical condition, 23% (*n* = 64) had underlying medical illness with non-communicable disease being the contributor.

Pertaining to family background characteristics, 77% (*n* = 214) had at least one dependent, 80.9% (*n* = 225) admitted to having family issues, and 64.7% (n = 180) felt that their workplace was far from their family.

### Quality of Life Among Participants Based on WHOQOL-BREF Domains

The average scores of all WHOQOL-BREF dimensions were approximately 70. The highest mean value (70.2) was observed for the physical health domain, followed by psychological health, social relationship, and environmental domains. Participants had low QOL in the domains of physical health (11.2%), psychological health (9.9%), social relationship (19.1%), and environment (14.4%).

### Explanatory Factors for QOL Among Participants Based on WHOQOL-BREF Domains

Table 2 presents both the crude and adjusted odds ratios (ORs) for variables in the WHOQOL-BREF P-QOL domain. All explanatory variables such as age, education level, marital status, monthly household income, campus location, work promotion, duration of current service, depression, anxiety, stress, medical illness, number of dependents, family issue, and workplace distance from family apart from job satisfaction indicated a significant influence on the P-QOL domain when the variables were regressed separately using the SLogR model (*p* < 0.05). Based on the MLogR analysis, three factors significantly affected the P-QOL domain. These were depression (OR = 3.49, 95% CI: 1.077–11.274), medical illness (OR = 1.36, 95% CI: 0.554–3.320), and number of dependents (OR = 1.96, 95% CI: 0.769–4.984). In other words, the three factors significantly affected the odds of having a good QOL in the P-QOL domain.

**Table 2:** Logistic regression analyses for the predictors of WHOQOL-BREF P-QOL domain.

**Predictors**	**Simple logistic regression**					**Multiple logistic regression**				
	**β**	***P*-value**	**OR**	**95% CI**		**Adj β**	***P*-value**	**Adj OR**	**95% CI**	
**Socio- demographic**										
Age	2.234	<0.001	9.33	4.693	18.560	0.145	0.774	1.16	0.429	3.119
Gender	2.094	<0.001	8.19	4.905	13.435	−0.001	0.998	1.00	0.447	2.231
Level of education	2.243	<0.001	9.42	5.871	15.118	−0.458	0.461	0.63	0.187	2.136
Marital status	1.768	<0.001	5.86	2.628	13.055	0.406	0.419	1.50	0.561	4.018
**Job factor**										
Household income	2.303	<0.001	10.00	3.578	27.949	−0.303	0.656	0.74	0.195	2.800
Job group	1.958	<0.001	7.09	4.580	10.966	−0.139	0.828	0.87	0.259	3.047
Campus location	2.357	<0.001	10.56	5.329	20.910	0.518	0.262	1.68	0.679	4.150
Work promotion	1.769	<0.001	5.87	3.393	10.143	−0.504	0.254	0.60	0.254	1.436
Current years of service	2.061	<0.001	7.86	4.505	13.703	−0.614	0.187	0.54	0.217	1.348
Job satisfaction	0.357	0.469	1.43	6.489	15.027					
**Psychological well-being**										
Depression	2.454	<0.001	11.62	6.592	20.468	1.248	0.037^*^	3.49	1.077	11.274
Anxiety	2.313	<0.001	10.10	5.274	19.342	−0.204	0.723	0.82	0.263	2.524
Stress	2.186	<0.001	8.90	5.606	14.130	−0.379	0.497	0.68	0.229	2.045
**Physical condition**										
Medical illness	2.218	<0.001	9.19	5.858	14.419	0.304	0.001^*^	1.36	0.554	3.320
**Family background**										
Family dependent	2.272	<0.001	9.70	6.122	15.370	0.672	0.045^*^	1.96	0.769	4.984
Family issue	2.079	<0.001	8.00	5.279	12.124	−0.056	0.923	0.95	0.303	2.946
Workplace far from family	2.024	<0.001	7.57	4.803	11.935	−0.348	0.476	0.71	0.271	1.839

* *Statistically significant at the 0.05 level*.

*Reference category: age: <45, gender: male, level of education: non-tertiary, marital status: married, household income: B40-M40, Job group: academician, campus location: urban, work promotion: yes, current years of service: <10 years, Job satisfaction: yes, depression: yes, anxiety: yes, stress: yes, medical illness: yes, family dependent: yes, family issue: yes, workplace far from family: yes*.

Table 3 illustrates both the crude and adjusted ORs for variables in the WHOQOL-BREF PSY-QOL domain. When regressed separately using the SLogR model (*p* < 0.05), all variables except for job satisfaction indicated significant influence on the PSY-QOL domain. However, further analysis based on MLogR proved that only four factors significantly affected the odds of having a good QOL in PSY-QOL. These factors were work promotion (OR = 0.28, 95% CI: 0.093–0.854), depression (OR = 19.25, 95% CI: 3.295–112.416), medical illness (OR = 3.89, 95% CI: 1.516–10.007), and number of dependents (OR = 0.19, 95% CI: 0.068–0.501). More specifically, lacking both depression and medical illness, being promoted, and having a dependent would increase the odds of having a good QOL in PSY-QOL domain.

**Table 3:** Logistic regression analyses for the predictors of WHOQOL-BREF PSY-QOL domain.

**Predictors**	**Simple logistic regression**					**Multiple logistic regression**				
	**β**	***P*-value**	**OR**	**95% CI**		**Adj β**	***P*-value**	**Adj OR**	**95% CI**	
**Socio-demographic**										
Age	2.110	<0.001	8.25	5.187	13.122	−0.192	0.752	0.83	0.250	2.724
Gender	2.225	<0.001	9.25	5.099	16.780	0.254	0.637	1.29	0.448	3.708
Level of education	2.132	<0.001	8.43	5.362	13.250	−0.669	0.267	0.51	0.157	1.670
Marital status	1.353	<0.001	3.80	1.893	7.626	−0.975	0.182	0.38	0.090	1.581
**Job factor**										
Household income	2.303	<0.001	10.00	3.578	27.949	−0.901	0.267	0.41	0.075	2.211
Job group	2.497	<0.001	12.14	5.618	26.255	0.160	0.834	1.17	0.262	5.250
Campus location	2.793	<0.001	16.33	7.163	37.244	0.753	0.297	0.41	0.075	2.211
Work promotion	1.621	<0.001	5.06	3.007	8.511	−1.269	0.025^*^	0.28	0.093	0.854
Current years of service	2.548	<0.001	12.78	6.484	25.182	−0.489	0.380	1.63	0.547	4.861
Job satisfaction	0.606	0.232	1.83	0.678	4.957					
**Psychological well-being**										
Depression	3.689	<0.001	40.00	14.831	107.882	2.957	0.001^*^	19.25	3.295	112.416
Anxiety	3.287	<0.001	26.75	9.859	72.578	−0.107	0.905	0.90	0.156	5.170
Stress	2.934	<0.001	18.80	9.952	35.514	0.410	0.519	1.51	0.434	5.237
**Physical condition**										
Medical illness	2.585	<0.001	13.27	7.850	22.422	1.360	0.051	3.89	1.516	10.007
**Family background**										
Family dependent	1.273	<0.001	3.57	1.975	6.460	−1.687	0.001^*^	0.19	0.068	0.501
Family issue	2.262	<0.001	9.60	3.822	24.114	−0.836	0.250	0.43	0.104	1.803
Workplace far from family	2.565	<0.001	13.00	6.027	28.042	−0.228	0.713	0.80	0.236	2.684

* *Statistically significant at the 0.05 level*.

*Reference category: age: <45, gender: male, level of education: non-tertiary, marital status: married, household income: B40-M40, Job group: academician, campus location: urban, work promotion: yes, current years of service: <10 years, Job satisfaction: yes, depression: yes, anxiety: yes, stress: yes, medical illness: yes, family dependent: yes, family issue: yes, workplace far from family: yes*.

Table 4 reveals both the crude and adjusted ORs for variables in the WHOQOL-BREF SR-QOL domain. All variables except for ethnicity and job satisfaction have a significant effect (*p* < 0.05) on the odds of having a good QOL when each of the variable was regressed separately. However, further analysis based on MLogR proved that only three factors significantly affected the odds of having a good QOL. The three factors were campus location (OR = 0.43, 95% CI: 0.227–0.864), depression (OR = 3.02, 95% CI: 1.595–5.731), and work promotion (OR = 0.48, 95% CI: 0.261–0.884). Simply put, the odds of having a good QOL in SR-QOL was greater among those who worked in urban areas, were promoted, and had no depression.

**Table 4:** Logistic regression analyses for the predictors of WHOQOL-BREF SR-QOL domain.

**Predictors**	**Simple logistic regression**					**Multiple logistic regression**				
	**β**	***P*-value**	**OR**	**95% CI**		**Adj β**	***P*-value**	**Adj OR**	**95% CI**	
**Socio-demographic**										
Age	1.730	<0.001	5.64	3.196	9.962	0.012	0.977	1.01	0.454	2.252
Gender	0.1512	<0.001	4.54	3.013	6.829	0.178	0.607	1.20	0.607	
Level of education	1.574	<0.001	4.82	3.334	6.979	0.245	0.503	1.28	0.624	2.616
Marital status	0.788	0.011	2.20	1.195	4.050	−0.576	0.261	0.56	0.206	1.535
**Job factor**										
Household income	2.054	<0.001	7.80	3.074	19.789	−0.128	0.828	0.88	0.277	2.793
Job group	1.636	<0.001	5.13	2.952	8.926	−0.801	0.098	0.45	0.174	1.159
Campus location	1.151	<0.001	3.16	2.015	4.935	−0.814	0.017^*^	0.44	0.227	0.864
Work promotion	0.985	<0.001	2.68	1.735	4.134	−0.734	0.018^*^	0.48	0.261	0.884
Current years of service	1.649	<0.001	5.20	3.222	8.391	0.008	0.984	1.01	0.483	2.102
Job satisfaction	0.118	0.808	1.13	0.434	2.916					
**Psychological well-being**										
Depression	1.974	<0.001	7.20	4.510	11.494	1.106	0.001^*^	3.02	1.595	5.731
Anxiety	2.110	<0.001	8.25	4.532	15.019	0.437	0.340	1.55	0.631	3.800
Stress	1.609	<0.001	5.00	3.441	7.266	−0.315	0.472	0.37	0.309	1.722
**Physical condition**										
Medical illness	1.533	<0.001	4.63	3.262	6.576	0.284	0.445	1.33	0.641	2.756
**Family background**										
Family dependent	1.017	<0.001	2.77	1.588	4.815	−0.137	0.769	0.87	0.350	2.175
Family issue	1.587	<0.001	4.89	2.378	10.014	−0.179	0.715	0.84	0.321	2.180
Workplace far from family	1.792	<0.001	6.00	3.407	10.565	0.677	0.069	1.97	0.948	4.091

* *Statistically significant at the 0.05 level*.

*Reference category: age: <45, gender: male, level of education: non-tertiary, marital status: married, household income: B40-M40, Job group: academician, campus location: urban, work promotion: yes, current years of service: <10 years, Job satisfaction: yes, depression: yes, anxiety: yes, stress: yes, medical illness: yes, family dependent: yes, family issue: yes, workplace far from family: yes*.

Table 5 signifies both the crude and adjusted ORs for variables in the WHOQOL-BREF E-QOL domain. All factors except ethnicity and job satisfaction significantly affected (*p* < 0.05) the odds of having a good QOL. However, further analysis based on MLogR showed that only age (OR = 3.216, 95% CI: 1.428–7.241), level of education (OR = 2.076, 95% CI: 1.088–3.962), depression (OR = 6.294, 95% CI: 2.893–13.695), work promotion (OR = 0.391, 95% CI: 0.195–0.780), and medical illness (OR = 2.072, 95% CI: 1.056–4.065) significantly affected the odds of having a good QOL in the E-QOL domain. Specifically, we can conclude that the odds of having a good QOL in the E-QOL domain was greater among those without medical illness and those who are older, are educated at a tertiary level, get promoted, and lack depression.

**Table 5:** Logistic regression analyses for the predictors of WHOQOL-BREF E-QOL domain.

**Predictors**	**Simple logistic regression**					**Multiple logistic regression**				
	**β**	***P*-value**	**OR**	**95% CI**		**Adj β**	***P*-value**	**Adj OR**	**95% CI**	
**Socio-demographic**										
Age	2.363	<0.001	10.63	5.147	21.934	1.168	0.005^*^	3.22	1.428	7.241
Gender	1.639	<0.001	5.15	3.190	8.314	0.438	0.279	1.55	0.701	3.427
Level of education	1.466	<0.001	4.33	2.472	7.597	0.730	0.027^*^	2.07	1.088	3.962
Marital status	1.897	<0.001	6.66	4.542	9.785	−0.464	0.441	0.63	0.194	2.044
**Job factor**										
Household income	1.705	<0.001	5.50	3.856	7.845	−0.489	0.472	0.61	0.162	2.362
Job group	1.997	<0.001	7.36	3.923	13.823	−1.002	0.084	0.36	0.118	1.144
Campus location	1.785	<0.001	5.96	3.902	9.104	0.059	0.893	1.06	0.451	2.495
Work promotion	1.138	<0.001	3.12	1.988	4.895	−0.940	0.008^*^	0.39	0.195	0.780
Current years of service	1.740	<0.001	5.70	3.657	8.871	−0.418	0.323	0.66	0.287	1.509
Job satisfaction	0.357	0.469	1.43	0.544	3.753					
**Psychological well-being**										
Depression	1.030	<0.001	2.80	1.845	4.248	1.840	<0.001^*^	6.29	2.893	13.695
Anxiety	2.555	<0.001	12.88	6.271	26.434	0.233	0.680	1.26	0.416	3.830
Stress	2.079	<0.001	8.00	5.136	12.462	−0.119	0.805	0.89	0.354	2.287
**Physical condition**										
Medical illness	1.853	<0.001	6.38	4.313	9.436	0.729	0.034^*^	2.07	1.056	4.065
**Family background**										
Family dependent	1.466	<0.001	4.33	2.313	8.118	0.047	0.929	1.05	0.373	2.947
Family issue	1.727	<0.001	5.63	2.652	11.932	−0.308	0.577	0.73	0.249	2.169
Workplace far from family	1.878	<0.001	6.54	3.647	11.721	0.089	0.847	1.09	0.443	2.698

* *Statistically significant at the 0.05 level*.

*Reference category: age: <45, gender: male, level of education: non-tertiary, marital status: married, household income: B40-M40, Job group: academician, campus location: urban, work promotion: yes, current years of service: <10 years, Job satisfaction: yes, depression: yes, anxiety: yes, stress: yes, medical illness: yes, family dependent: yes, family issue: yes, workplace far from family: yes*.

## Discussion

Our study found that the level of QOL for each domain was approximately the same to that of a similar population of university staff from Brazil (11). However, the level of QOL among university staff was considerably higher in every domain compared to the general population in our country (52). A key contributing factor was the general population's socioeconomic background. Since this study had focused on university staff who were all gainfully employed, they had a stable income, as opposed to the general population that included some who were unemployed and have a lower socioeconomic background.

This study affirmed that depression was significantly associated with all four domains of the QOL. This finding was consistent with previous literature showing lower QOL in individuals with major mental illnesses, particularly depression (16, 53–55). Globally, depression was among the leading cause of disability with significant psychosocial and occupational impairment (56). Apart from that, depression has been found to promote the development of chronic medical illnesses (57), resulting in further disability and lower QOL. Furthermore, previous studies had suggested that factors related to depressive symptoms such as severity, chronicity, number of relapses, and residual symptoms may be associated with a lower QOL (53, 57).

The presence of medical illness was found to have negatively impacted three domains of the QOL, which were physical, psychological health, and environmental. This result was in line with past literature which suggested that chronic medical illness contributed to reducing QOL (58–60). Likewise, having medical illnesses affected an individual, physically leading to occupational impairment and significant psychological distress, thereby reducing the level of QOL. However, some studies had showed that factors such as the individuals' acceptance of the illness, adaptive coping strategies, and good psychosocial support can improve QOL in persons with medical comorbidity (59–61).

Pertaining to the location of campuses, those working in urban areas had better QOL than those in suburban areas (62–64). Urban cities are often the main areas of economic, social, and political growths, which provide various opportunities to their dwellers, especially among the working class (62). Additionally, their superior living conditions, with easy to reach amenities as well as specialized healthcare facilities, abundance of education opportunities, and availability of more specialized job prospects rendered greater QOL.

Our result has also shown that university staff with higher levels of education enjoyed better QOL as compared to those with lower levels of education. Formal education imparts knowledge, cultural values, and life skills that are deemed essential in one's life (27, 28). It is also closely linked to healthier occupational trajectory, income opportunities, and future life opportunities, which would positively affect standard of living and QOL. Moreover, in a study by Eriksson et al. involving human immunodeficiency virus (HIV)-infected persons, a significant relationship was found between higher levels of education and better QOL (65).

Certainly, lack of work promotion opportunities would negatively impact the staff's QOL. Promotion opportunities were indeed essential factors linked to superior quality of work life, positive work experience, and job satisfaction (2, 33, 34, 65). Besides being an incentive to maintain employees' motivation and productivity, they often offered substantial wage increment and job security, which were vital for good QWL (66). Furthermore, better promotion exercises among university staff in our study were associated with good QOL, a finding that aptly corroborated a cross-sectional survey among 386 teachers from a public higher education organization situated in the Central-West Region of Brazil (41).

Aside from that, having dependents was associated with a lower QOL. A larger household size (67, 68) with more dependents (68, 69) placed a higher burden on financial status and poorer standards of living, which then lowered the QOL. In contrast, a smaller household size had better economic status and QOL.

Although literature had described reduced QOL in relation to anxiety disorders such as panic disorder (14–16), generalized anxiety disorder (16–18), and social anxiety disorder (14), our current findings did not show significant association between anxiety symptoms and QOL. This could be confounded by the severity of symptoms (14, 17), an individual's coping method (18), and availability of support (14), which were not explored in this research.

We acknowledge several limitations in this study. Firstly, the study was conducted in a selected local university wherein most of the staff members were Malays. Thus, it may not be an accurate representation, and this limits its generalization. Furthermore, the percentage of staff working in the urban and suburban areas was neither proportionate nor stratified according to the actual ratio. Lastly, the study design's cross-sectional nature might not demonstrate the cause-and-effect relationship between the variables.

## Conclusion

Apart from focusing on physical health, the present study highlighted the need for an early detection, mental health promotion, and provision of mental health services to those in need, especially among university staff with depression, to improve their QOL. University administrators and policymakers may also consider this issue in their future planning and allocate resources accordingly.

## Data Availability Statement

The data that support the findings of this study are available upon formal request. The data are not publicly available due to privacy concerns.

## Ethics Statement

Ethical approval was obtained from the Institutional Ethics Committee (600-IRMI-5/1/6-REC/398/18). The patients/participants provided their written informed consent to participate in this study.

## Author Contributions

SR, MN, and HN contributed to conception and design of the study. SR organized the database. YA performed the statistical analysis. MN wrote the first draft of the manuscript. SR, MN, SA, and YA wrote sections of the manuscript. All authors contributed to manuscript revision, read, and approved the submitted version.

## Conflict of Interest

The authors declare that the research was conducted in the absence of any commercial or financial relationships that could be construed as a potential conflict of interest.

## Publisher's Note

All claims expressed in this article are solely those of the authors and do not necessarily represent those of their affiliated organizations, or those of the publisher, the editors and the reviewers. Any product that may be evaluated in this article, or claim that may be made by its manufacturer, is not guaranteed or endorsed by the publisher.
